# Decreased coagulation kinetics is associated with high blood loss in patients with end-stage liver disease undergoing liver transplantation

**DOI:** 10.1186/cc14425

**Published:** 2015-03-16

**Authors:** M Popescu, D Tomescu

**Affiliations:** 1 Carol Davila University of Medicine and Pharmacy, Bucharest, Romania

## Introduction

Our aim was to assess hemostasis, using ROTEM, in patients with end-stage liver disease (ESLD) undergoing liver transplantation (LT) and to develop a predictive model for patients prone to high intraoperative blood loss.

## Methods

We retrospectively analyzed 122 patients who underwent LT between January and December 2013 in a single national center. Patients with acute liver failure or incomplete data were excluded. Demographic data, severity of liver disease assessed by MELD score (model for ESLD), presence of portal vein thrombosis, and laboratory data were recorded preoperatively. We performed concomitant ROTEM assay and standard coagulation tests (prothrombin time (PT), International Normalized Ratio (INR), fibrinogen) 1 hour before surgery and 15 minutes after the neohepatic phase. Intraoperative blood loss was recorded. High blood loss was defined as loss of one blood volume during surgery. Correlation between recorded data standard ROTEM parameters and derived thrombodynamic ROTEM parameters (potential index (TPI), maximum velocity of clot formation (MaxV), time to MaxV (MaxVt), AUC) were analyzed using SPSS 19.0.

## Results

After applying exclusion criteria, 72 patients were analyzed with mean age of 54.5 years (SD 11.6) and a median MELD score of 17.4 (7 to 34). Preoperative MCE correlated with age (*P *= 0.044, 95% CI (-7.50, -0.12)) and MELD score (*P *= 0.009, 95% CI (-37.21, -6.69)), but not with PT (*P *= 0.557) or INR (*P *= 0.623). MaxV correlated with fibrinogen level (*P *= 0.005, 95% CI (0.01, 0.05)) and AUC correlated with age (*P *= 0.034, 95% CI (-257.74, -11.91)) and MELD score (*P *= 0.01, 95% CI (-1,233.14, -215.33)). Patients with portal vein thrombosis had an increase in InTEM CFT (*P *= 0.002, 95% CI (77.98, 317.97)) and MaxVt (*P *= 0.03, 95% CI (5.53, 105.63)). No correlation was found between preoperative ROTEM parameters and intraoperative blood loss. We calculated ΔMaxV, ΔMaxVt and ΔAUC as the mathematical difference between preoperative and intraoperative MaxV, MaxVt and AUC. High blood loss correlated with ΔAUC (*P *= 0.005, 95% CI (15.69, 61.03)), ΔMaxV (*P *= 9 = 0.002, 95% CI (-20,413, 6,392)) and ΔMaxVt (*P *= 0.008, 95% CI (15.69, 61.07)).

## Conclusion

MELD score correlated with a decrease in MaxV and AUC on preoperative ROTEM but not with INR. Patients with portal vein thrombosis have increased InTEM CFT and MaxVt. High blood loss was associated with a decrease in thrombodynamic parameters, but no correlations were found between blood loss and standard ROTEM parameters.

